# A patient with patent foramen ovale associated ischemic stroke combined with hemorrhagic transformation: A case report

**DOI:** 10.1097/MD.0000000000042520

**Published:** 2025-05-23

**Authors:** Yan Yang, Han Luo

**Affiliations:** a Department of Neurology, Shenzhen University General Hospital, Shenzhen, Guangdong, P. R. China; b Department of Neurology, Shenzhen Longhua District Central Hospital, Shenzhen, Guangdong, P. R. China.

**Keywords:** patent foramen ovale, TCD foaming test, hemorrhagic transformation, cryptogenetic stroke

## Abstract

**Rationale:**

Magnetic resonance imaging (MRI) of patients with patent foramen ovale (PFO)-associated stroke often shows scattered, multiple ischemic lesions, mostly located in the cerebral cortex or vertebrobasilar artery. PFO occlusion is recommended for patients with large right-to-left shunt signals (>4 mm), moderate to large right-to-left shunts, and multiple ischemic lesions on imaging examination if an embolic event is suspected.

**Patient concerns:**

A 66-year-old male was admitted to the hospital with dizziness and blurred vision for 16 hours. No other symptoms or signs were observed.

**Diagnoses:**

The transcranial doppler bubble test showed a rain curtain-like right-to-left shunt in a resting state, and ultrasound contrast indicated a right-to-left shunt at the level of the atria. MRI indicated a patient infarction site in the right temporal lobe and occipital cortex, and hemorrhage within the infarcted lesion.

**Interventions:**

Surgery for PFO occlusion was performed on June 8, 2023.Two subcutaneous injections of low-molecular-weight heparin were administered postoperatively for anticoagulant therapy with 12-hour intervals between 2 shots, and aspirin and clopidogrel were administered for antiplatelet therapy.

**Outcomes:**

The patient’s condition improved significantly after the operation. During the 3-month follow-up, postoperative cardiac ultrasonography showed that the shape and position of the occluders were normal, and no obvious shunt was found at the atrial level after PFO closure. Dizziness and blurred vision in both eyes returned to normal. The patient’s condition was stable, with no symptoms of neurological deficits. As a result, clopidogrel was discontinued.

**Lessons:**

The transcranial doppler bubble test, a key test for clinical screening of PFO-related stroke, is easy to operate, highly sensitive, less costly, and causes less pain. Combined with ultrasound contrast, it can rapidly identify whether a PFO exists.

## 1. Introduction

The clinical manifestations of patent foramen ovale-associated stroke (PFO-AS) are dramatically different, and its embolic characteristics differ greatly from those of other types of cardiogenic embolisms, making it highly likely to be misdiagnosed or a missed diagnosis could occur. The transcranial Doppler (TCD) bubble test combined with ultrasound contrast of the right heart can rapidly identify whether patent foramen ovale (PFO) exists. Its easy operation and high sensitivity make it widely applicable in the early diagnosis of PFO-AS. In this report, we discuss a patient with PFO-associated ischemic stroke combined with hemorrhagic transformation (HT) to provide a basis for the diagnosis and treatment of this type of ischemic stroke.

## 2. Case presentation

**Figure 1.**
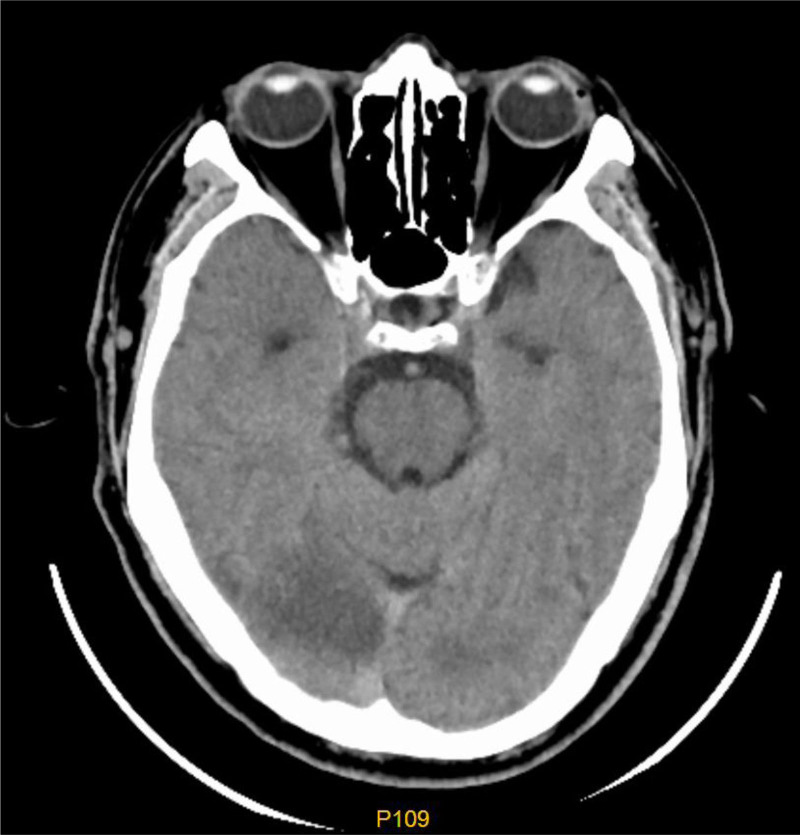
Plain CT scan of the brain (May 26, 2023) suggests cerebral infarction in the right occipital lobe. CT = computed tomography.

A 66-year-old male admitted to Shenzhen University General Hospital on May 27, 2023, for dizziness and blurred vision for 16 hours. The patient had a sudden onset of dizziness and blurred vision in both eyes, accompanied by nausea and vomiting 16 hours prior (08:30 on May 27), without slurred speech, numbness or weakness in the limbs, headache, or unsteady walking. His symptoms were not relieved, and he went to the Emergency Department of the Shenzhen University General Hospital at 22:30, where he had his blood pressure measured at 170/102 mm Hg, and his emergency cranial CT showed cerebral infarction in the right occipital lobe. Therefore, he was admitted to the Neurology Department. His medical history included hyperlipidemia, but he did not have hypertension, diabetes mellitus, or cardiac disease. He had occasional drinking, but denied a long-term smoking history. Admission examination showed a blood pressure of 165/99 mm Hg in the right upper limb, 160/91 mm Hg in the left upper limb, and a heart rate of 65 beats/min. He was conscious and could speak normally. The bilateral pupil diameter was 3 mm and the eyes were sensitive to direct and indirect light reflexes. Left upper quadrant vision loss was observed in both eyes, and the bilateral frontal lines and nasolabial groove were symmetric. The tongue was centered, and pharyngeal reflexes, limb muscle strength, and muscle tension were normal. No ataxia or sensory disturbances were observed. Tendon reflexes were symmetrical. The bilateral pathological findings and signs of meningeal irritation were negative. Other examinations included routine blood tests and tests of liver function, renal function, coagulation function, thyroid function, blood sugar level, glycosylated hemoglobin A1c, cholesterol level, and low-density lipoprotein levels, all of which were normal. Plain computed tomography (CT; 2023-5-26) of the brain revealed cerebral infarction in the right occipital lobe (Fig. 1).

**Figure 2.**
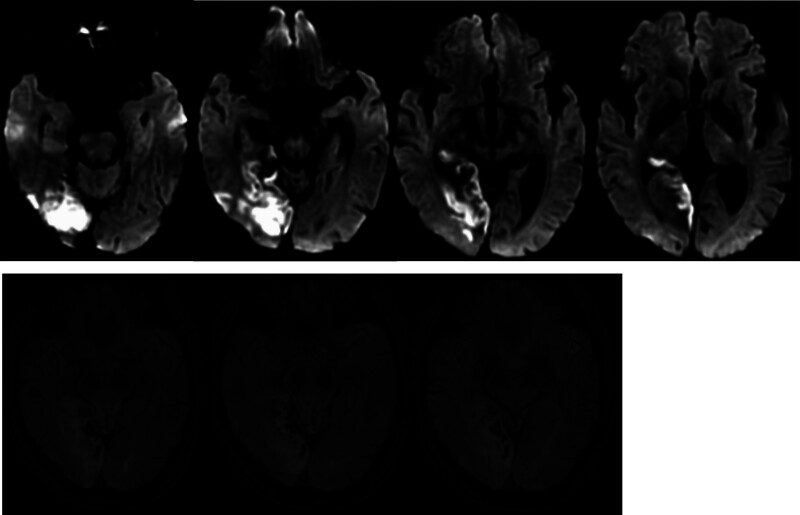
(A) DWI sequence shows hyperintensity in the right temporo-occipital lobe. (B) SWI sequence indicates abnormal signals in the right temporo-occipital lobe, so multiple microhemorrhagic lesions are suspected. DWI = diffusion weighted imaging, SWI = susceptibility weighted imaging.

**Figure 3.**
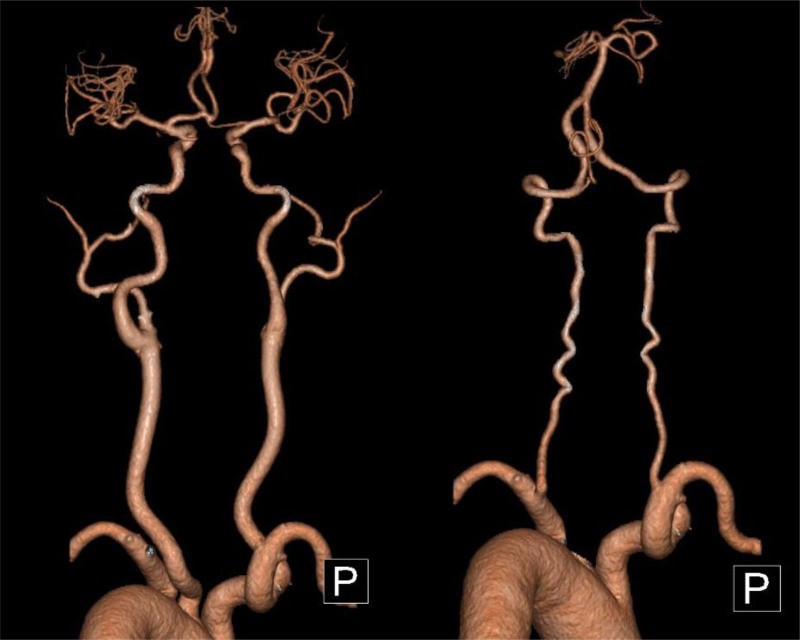
CTA of the brain and neck does not show intracranial and extracranial large artery stenosis. CTA = computed tomography angiography.

**Figure 4.**
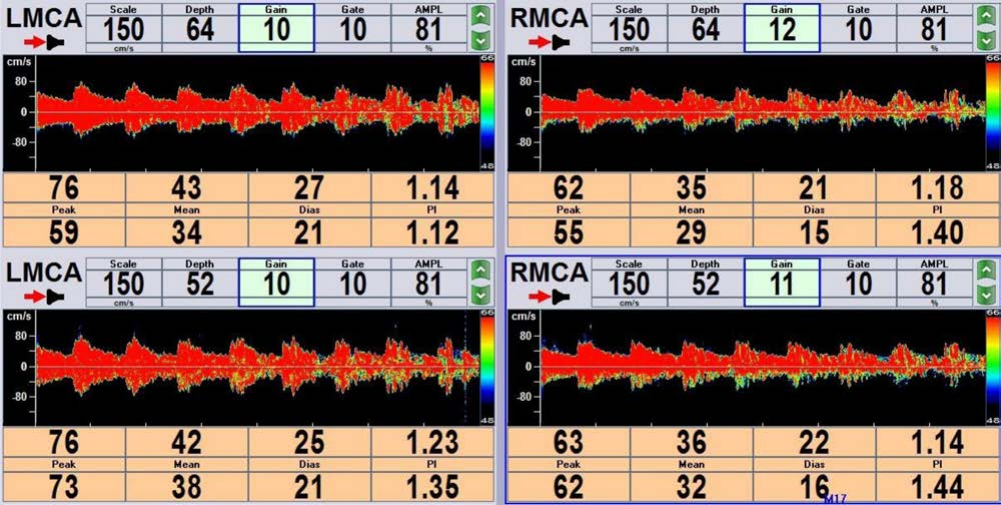
Bubble test monitors the bilateral middle cerebral arteries and rain curtain-like microbubble signals (right-to-left shunt) are found in a calm respiratory state.

**Figure 5.**
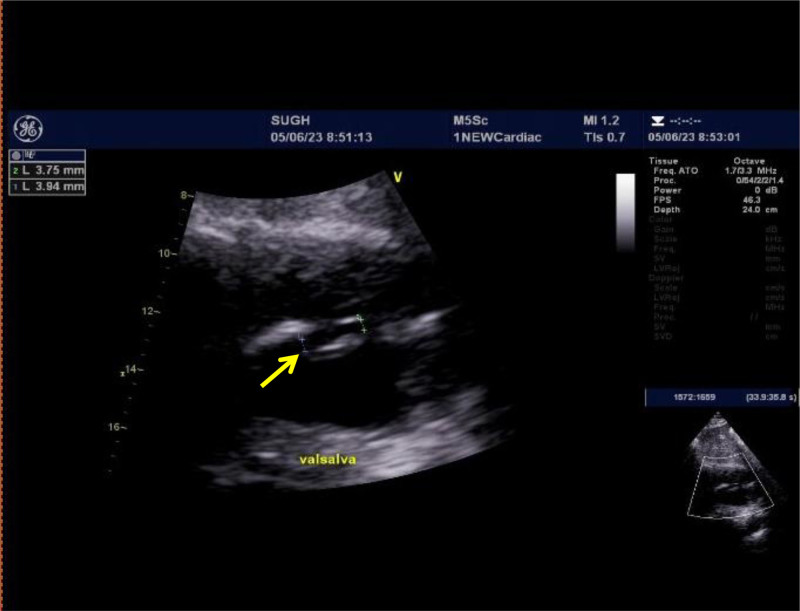
Cardiac ultrasound shows separation of the primary and secondary septum of the atrial septum after the Valsalva maneuver, with about 3 mm in width. So PFO is suspected. PFO = patent foramen ovale.

After admission, aspirin and clopidogrel were administered to prevent platelets and atorvastatin from lowering lipid levels and promoting blood circulation. Brain MRI (May 28, 2023) showed hemorrhagic cerebral infarction in the early stage of the right occipital and temporal lobes. Susceptibility weighted imaging revealed multiple less severe bleeds in the right occipital lobe and temporal lobe (Fig. 2). Multiple White matter hyperintensities in the bilateral frontal and parietal subcortices and around the lateral ventricles (Fazekas grade 3). Computed tomography angiography of the brain or neck and cranial perfusion (May 28, 2023) showed no abnormalities (Fig. 3). A phase II hypoperfusion was observed in the right occipital infarct. Cardiac ultrasonography (May 28, 2023) suggested left atrial enlargement, widening of the ascending aorta, mild regurgitation of the bicuspid and tricuspid valves, and normal systolic function of the left ventricle. The conventional electrocardiogram (May 29, 2023) was normal, and an ambulatory electrocardiogram (May 30, 2023) showed episodic atrial and ventricular premature beats. The TCD bubble test (June 1, 2023) showed a large right-to-left shunt in the calm respiratory state, and no Valsalva maneuver was required (Fig. 4). Ultrasound contrast of the right heart (June 5, 2023) suggested a right-to-left shunt at the level of the atria, and PFO was suspected (Fig. 5). Based on the patient infarction site in the right temporal lobe and occipital cortex, hemorrhage within the infarcted lesion, and risk of paradoxical embolism score of 6, PFO-associated cerebral infarction was highly suspected. A consultation with doctors from the Department of Cardiovascular Medicine was held and the patient was transferred to the Department of Cardiovascular Medicine for occlusion treatment. After preoperative examinations, surgery for PFO occlusion was performed on June 8, 2023. Intraoperative transesophageal ultrasonography showed that the primary and secondary septa of the interatrial septum deviated, with a maximum separation of 4 mm and a tunnel length of approximately 7 to 8 mm, suggesting that the foramen ovale was not closed. Postoperative cardiac ultrasonography (June 9, 2023) showed that the shape and position of the occluders were normal, and no obvious shunt was found at the atrial level after PFO closure. Two subcutaneous injections of low-molecular-weight heparin were administered postoperatively for anticoagulant therapy with 12-hour intervals between two shots, and aspirin and clopidogrel were administered for antiplatelet therapy.

## 3. Discussion

### 3.1. Is patent foramen ovale a perpetrator or bystander in cases of cryptogenic stroke with patent foramen ovale?

Stroke is the second leading cause of death worldwide; ischemic stroke accounts for 70% of cases and has a high risk of recurrence.^[1,2]^ Ischemic stroke is a heterogeneous disease with many causes that can lead to vascular occlusion and cerebral infarction. According to the trial of org 10172 in acute stroke treatment classification, if the available tests do not identify the cause, cryptogenic stroke is highly suspected and accounts for approximately 10% to 40% of cases.^[3,4]^ Patent foramen ovale (PFO) usually refers to a condition in which the primary and secondary septa of the atrial septum of the heart are not anatomically close between birth and the age of 3 years old. An abnormal anatomical passage remains between the two, and this passage is referred to as the PFO.^[5]^ In recent years, widely applied and improved PFO examination techniques have resulted in more cases of cryptogenic stroke being diagnosed with PFO. In addition, PFO can be confirmed after excluding other possible causes of ischemic stroke. Therefore, some scholars have called for PFO-AS to be included in the etiological classification of ischemic stroke subtypes.^[6]^ It was previously believed that further screening for PFO should be performed only in patients without risk factors for cerebrovascular disease. Approximately 25% to 30% of adults have PFO,^[5,7]^ it is not clear whether PFO is the cause of cryptogenic strokes or a bystander. However, this may result in a missed diagnosis of ischemic stroke owing to PFO. Yiwen Zhang et al^[8]^ reported a case of a 60-year-old man with a combination of hypertension, diabetes, smoking, carotid plaque, risk factors for the siphon segment of the bilateral internal carotid artery, and repeated cerebral ischemia. He was misdiagnosed with cerebral small vessel disease and was administered antiplatelet and statin to prevent plaque and risk factors; however, recurrence still occurred. PFO was detected by ultrasound contrast of the right heart and TCD bubble test. After treatment with PFO occlusion and 20 mg of rivaroxaban for anticoagulation, the patient did not have recurrent stroke. Therefore, even with common risk factors for cerebrovascular disease such as hypertension, diabetes, and cerebrovascular stenosis, the possibility of PFO cannot be completely ruled out as we usually think.

### 3.2. Embolic features of patent foramen ovale-associated stroke

In the case of PFO-AS that we reported, the blood pressure was elevated on admission, but it gradually reduced to normal without taking any medication. Therefore, increased blood pressure on admission was considered to be a result of stress. Although the patient was older than 60 years of age, there were no severe risk factors for cerebrovascular diseases. After ruling out the possibility of intracranial and extracranial large artery stenosis and cardiac embolisms, such as atrial fibrillation and cardiac valvular disease, the TCD bubble test showed a rain curtain-like right-to-left shunt in a resting state, and ultrasound contrast indicated a right-to-left shunt at the level of the atria. So PFO was suspected.^[9]^ Therefore, stroke is suspected to be related to PFO. Although there are references indicating suggest that MRI imaging of patients with PFO-AS may shows scattered, multiple ischemic lesions, mostly located in the cerebral cortex, subcortical or vertebrobasilar artery,^[10,11]^ these are only small sample studies and there is currently no consensus. In contrast to atrial fibrillation, which is also a type of embolic cerebral infarction, involves malignant cerebral infarction in a large part of one cerebral hemisphere, small infarcted lesions caused by PFO-associated embolic strokes are often scattered in the cortex or subcortex, which is hypothesized to be linked to differences in the composition and source of emboli. The venous system is often the source of emboli in PFO, whereas the source of emboli in atrial fibrillation is from the left atrium, and large arteries are often embolized.^[12]^ Cranial MRI showed infarcts in the right occipital and right temporal cortices with HT, which is consistent with the symptoms of embolic cerebral infarction. Ischemic stroke combined with HT is commonly found in the gray matter of the brain, particularly in the cerebral cortex. The cerebral cortex has many collateral circulations, aggravating reperfusion injury in the ischemic region and leading to HT.^[13]^

### 3.3. Preventive measures for patent foramen ovale-associated stroke

It is more effective, and medication or interventional occlusion to treat PFO-related stroke has not been reported. With further in-depth research, the results of the 10-year follow-up of the RESPECT study in the United States showed that PFO occlusion with an Amplatzer occluder reduced the risk of stroke recurrence in patients with stroke combined with PFO of unknown cause. This benefit was greater than that in patients receiving treatment with drugs.^[14]^ Therefore, Amplatzer occluders have been approved in the United States for the prophylactic treatment of PFO-related stroke. The Chinese Expert Consensus on Prophylactic Occlusion of PFO points that PFO occlusion is recommended for patients with large PFOs (>4 mm), moderate to large right-to-left shunts, and multiple ischemic lesions on CT or MRI if an embolic event is suspected; for patients who do not undergo PFO occlusion, antithrombotic therapy with drugs such as aspirin or anticoagulant is recommended.^[15]^ In conclusion, PFO occlusion aims to prevent the recurrence of PFO-related strokes. Surgery requires consultation of doctors from the divisions of neurology and cardiovascular medicine to ensure that occlusion is effective. Given the fact that the venous system of elderly patients is more likely to cause thrombus than that of younger patients and that the patients in this cohort had PFO-related stroke, PFO occlusion was performed after consultation with doctors from the divisions of neurology and cardiovascular medicine and strong demands from the patients and their relatives.

## 4. Conclusions

The TCD bubble test, a key test for clinical screening of PFO-related stroke, is easy to perform, highly sensitive, less costly, and causes less pain. It can be combined with ultrasound contrast to identify whether a patient has developed PFO. Using ultrasound contrast of the right heart alone is highly likely not to detect PFO because it is affected by the patient respiratory movements.^[16]^ Although transesophageal ultrasound has been deemed the gold standard for screening for PFO, it is not widely applied because it is invasive, painful, and risky.^[17]^ Some studies have reported that the transcranial Doppler bubble test, as an indirect screening method for PFO, has a sensitivity of 96%, specificity of 80%, and 90% consistency with the findings from transesophageal ultrasound.^[18]^ In conclusion, PFO is a common anatomical developmental abnormality and its pathogenicity in ischemic stroke cannot be ignored. The TCD bubble test can be used as an auxiliary examination for PFO.

## Acknowledgments

We would like to express our gratitude to the patient family members for granting permission to use the patient clinical data in this paper and for the publication of this research.

## Author contributions

**Writing – original draft:** Yan Yang.

**Writing – review & editing:** Han Luo.
